# Persistent Paradoxical Reaction to Midazolam despite General Anesthesia with Dexmedetomidine

**DOI:** 10.1155/2024/4152422

**Published:** 2024-03-26

**Authors:** Sarah Park, Mariam Ibrahim, Augusto Torres

**Affiliations:** ^1^Case Western Reserve University School of Medicine, 9501 Euclid Avenue, Cleveland, OH 44106, USA; ^2^Department of Anesthesiology, MetroHealth Medical Center, 2500 MetroHealth Drive, Cleveland, OH 44109, USA

## Abstract

Midazolam is a widely used benzodiazepine due to its rapid onset of action and relatively safe side effect profile. It is used for sedation, anxiolysis, and induction of general anesthesia. However, in <1% of instances, it may cause a paradoxical excitement: agitation, restlessness, myoclonus, stiffening of the limbs, and aggression. We report a case report in which preoperative administration of midazolam caused onset of the aforementioned symptoms that were not attenuated by general anesthesia with dexmedetomidine. This case report aims to create awareness about the rare adverse reactions of midazolam and prepare clinicians to manage these situations.

## 1. Introduction

Midazolam is a rapid-onset, short acting benzodiazepine commonly used in various settings to provide sedation and anxiolysis before general anesthesia due to its relatively safe and predictable profile. However, paradoxical reactions to midazolam can occur idiosyncratically, consisting of excitatory manifestations (agitation, confusion, delirium, inconsolable hysteria, aggression, and restlessness) and motor disturbances (dystonia, dyskinesia, tremor, and athetoid movements) [1, 2]. Dexmedetomidine is often used to reduce agitation and provide sedation. We report one such case in which intravenously administered midazolam caused a paradoxical reaction that persisted despite general anesthesia with dexmedetomidine.

## 2. Case Presentation

A 63-year-old female, weighing 106 kg, sustained a mechanical fall secondary to feeling lightheaded and presented immediately to the hospital. She was found to have right type VI tibial and fibular fractures. Her past medical history was pertinent for sleep apnea, hypothyroidism, hyperlipidemia, Roux-en-Y gastric bypass, anxiety, depression, and tardive dyskinesia. Her medications included levothyroxine, atorvastatin, sertraline, quetiapine, Atarax, gabapentin, and Cogentin, all of which she was taking regularly. She presented for an urgent open reduction and internal fixation of her right tibia and fibula to the perioperative area. Her neurological exam on preoperative assessment revealed upper extremity intention tremors, which were chronic per the patient's medical records and being fully alert and oriented to time, place, and person.

She received 2 mg of midazolam IV before she was taken back to the operating room due to being especially anxious. As she was entering the operating room, an acute mental status change occurred. She became noticeably agitated and disorientated and developed diffuse tremulousness with myoclonus. Due to the urgent nature of the surgery and the possibility of general anesthesia along with dexmedetomidine potentially ameliorating the reaction, the decision was made to proceed with general anesthesia without reversal of the reaction with flumazenil. She underwent general anesthesia with 100 mg propofol, fentanyl, a dexmedetomidine infusion, and rocuronium for maintenance. Dexmedetomidine was added to reduce the agitation noted prior to induction. She was extubated after reversal with sugammadex after a 2.75-hour long surgery.

Postoperatively, the patient had tangential thoughts, remained agitated and anxious, and was only oriented to name. On exam, she had paratonia, asterixis, and diffuse myoclonus of her bilateral upper extremities and left lower extremity. (Her right lower limb was bandaged.)

Due to these findings, the neurology service was consulted in the post anesthesia care unit. The exam was not consistent with serotonin syndrome or extrapyramidal syndrome. TSH, electrolytes, ammonia, and gabapentin levels were within normal limits. Her urine toxicology screen was negative, and a noncontrast head CT scan was also negative for any pertinent findings. Fat embolism was less likely due to lack of clinical symptoms, such as petechial rash, hypoxemia, retinal changes, or jaundice, and acute presentation after midazolam was administered. The patient did not meet Gurd's criteria for fat embolism. After the differential diagnoses were narrowed, it was deemed likely that the patient had a toxic-metabolic encephalopathy potentially linked to a paradoxical reaction to midazolam. Neurology recommended decreased doses of sertraline and quetiapine and discontinuation of cogentin. It was unlikely that these medications interacted, but doses of sertraline and cogentin were altered to prevent their contribution to the patient's delirium. Quetiapine was decreased because it could worsen myoclonus. However, flumazenil to treat the patient's paradoxical reaction to midazolam was not recommended because there was a potential to aggravate her toxic encephalopathy with another drug or cause adverse side effects.

Over the course of the next few days, the patient started to become oriented to place and person with eventual resolution of her delirium. Her myoclonus self-resolved by postoperative day 5 and she was discharged with return of her baseline mental status preoperatively.

## 3. Discussion

Midazolam is a common benzodiazepine used in up to 75% of sedations in the United States due to its relative safety profile [3]. However, rare, unexpected side effects are possible, with the most common being a paradoxical reaction consisting of a mixture of dyskinetic motor disturbances (tremors, dyskinesia, and dystonia) and mental agitation (delirium, aggression, and anxiety) [4, 5]. This reaction can occur in any patient, with incidence in children as young as 1–3 years of age [6]. Risk factors include younger age, higher dose of midazolam, having a psychiatric background, alcohol abuse, and genetic background [7]. Our patient showed an acute change in mental status reflected by increased inattention and disorientation, along with diffuse tremors with myoclonus shortly after receiving 2 mg of IV midazolam for anxiolysis.

There have been multiple postulations concerning what might cause midazolam's paradoxical reaction. Benzodiazepines stimulate the effect of gamma-aminobutyric acid (GABA-A) in the ascending reticular activating system [8]. One theory is that genetic variability in benzodiazepine receptor affinity or density can profoundly alter the pharmacodynamics of this drug. Benson et al. showed that even a single mutation in the GABA receptor subunit caused GABA receptors to react differently to diazepam [9, 10]. Patients with such mutations could be more sensitive to benzodiazepine side effects, requiring a lower dose than expected, or have a different reaction to the drug altogether. A second theory is that midazolam could cause a serotonin imbalance by reducing serotonin turnover, which has been linked to symptoms such as disinhibition and aggression [11, 12]. Third, there have been implications that benzodiazepines are linked with central cholinergic activity, which could explain symptoms such as dystonia and tremors in the paradoxical reaction. This may be further supported by the use of physostigmine, an anticholinesterase inhibitor, as an antidote to these paradoxical reactions before the modern use of flumazenil for this same purpose [12]. Physostigmine was not used in our patient due to its inconsistent reversal of midazolam's paradoxical reaction and adverse side effects, such as dyspnea, bradycardia, nausea, vomiting, and epigastric pain. Finally, midazolam is metabolized by cytochrome CYP3A4 enzymes. Various medical conditions and drug-drug interactions can affect the activity of these enzymes, which may alter the metabolism of the drug and cause prolonged effects [13]. Further study is needed to better predict what type of patients may have higher propensities for unexpected effects of midazolam.

Current literature on the best reversal agent for midazolam's paradoxical reaction revolves around using flumazenil [14–16]. Other studies have also successfully used physostigmine, ketamine, and haloperidol to reverse this reaction [7, 17–19]. However, our case is one of the few where a patient's paradoxical reaction was not reversed with any type of agent due to the need for urgent surgical management and the hope that general anesthesia with dexmedetomidine would eventually resolve this reaction.

Our case report highlights a few points: First, general anesthesia was insufficient to reverse or attenuate midazolam's paradoxical reaction in our patient. Although this reaction is neither fatal nor prevalent, treatment with a reversal agent should still be considered due to potential consequences from lingering symptoms such as prolonged anterograde amnesia postoperatively leading to patient distress, bodily harm from aggressive behavior, distress of caregivers or family, and a prolonged postoperative course. Anesthesiologists should consider giving an appropriate reversal agent promptly upon seeing these effects or consider treatment postoperatively. Because flumazenil has a shorter half-life than midazolam, it may be necessary to give a repeat dose or even a continuous infusion if symptoms persist [12, 13].

Second, a dexmedetomidine adjunct may be inadequate to alleviate agitation from midazolam's paradoxical effect. Dexmedetomidine is a versatile drug used in both the ICU and surgical setting due to its predictable effect profile from extreme selectivity of alpha-2 over alpha-1 receptors [20]. It has many appealing properties such as causing little to no respiratory depression, which was particularly useful in our morbidly obese patient with sleep apnea who was susceptible to narcotic-induced respiratory depression, and generally having anxiolytic, sympatholytic, sedative, and analgesic properties [21]. In this case, in addition to being used as an adjunct surgically, dexmedetomidine was used as a potential strategy to ameliorate agitation from the patient's paradoxical effect. However, despite dexmedetomidine's sedating effects, the patient was still agitated and showed paradoxical symptoms postoperatively, leading to the last point.

Midazolam's paradoxical effect can persist for an extended period of time postoperatively and contribute to a toxic-metabolic state. Because the differential diagnosis for postoperative delirium is extensive, considering a persistent paradoxical effect from midazolam used preoperatively may be a diagnosis of exclusion. Our patient had inattention, disorientation, and diffuse myoclonus postoperatively. She exhibited similar symptoms preoperatively after receiving midazolam. These symptoms eventually resolved.

Necessary efforts were made to assess the patient's symptoms postoperatively and narrow her differential diagnosis: Noncontrast head CT and urine toxicology screen were negative for pertinent findings, and screenings for electrolytes, ammonia, magnesium, and TSH all returned normal within normal limits. In addition, serotonin syndrome was unlikely because her exam was negative and she was not on multiple serotonergic medications, except sertraline that was reduced in dose just to be extra safe. Fat embolism was considered due to the patient's clinical context of having long bone fractures and her reaction not being resolved with general anesthesia. However, this differential was less likely because the patient lacked other clinical symptoms of fat embolism, such as hypoxemia, petechial rash, jaundice, retinal changes, or vital instability. Intraoperatively, the patient had no desaturations or hypotensive episodes, ruling out cerebral hypoxia, and maintained normal intraoperative glucose levels. Delirium is also sometimes attributed to pain. However, our patient did not have visible signs of significant pain (such as grimacing, yelling, or reaching for her leg) and had access to intravenous hydromorphone PRN (Dilaudid IV push) postoperatively, making pain a less likely etiology of her symptoms. Finally, although uncommon in adults, emergence delirium could have been a potential differential that was thought less likely because the patient's postoperative symptoms were very similar to those exhibited preoperatively following administration of midazolam. In addition, emergence delirium is typically short-lived, while this patient's agitation lasted for days. With this exclusion of multiple differentials, it seemed likely that her symptoms were tied with midazolam.

Despite this potential etiology, flumazenil was not used to reverse these symptoms postoperatively in consultation with neurology, despite being available for use, due to risk of drug interactions (the patient was regularly taking many medications), hope that dexmedetomidine or general anesthesia could ameliorate the reaction, and risk of causing other adverse side effects [22]. Because delirium is considered a generally reversible condition, it was deemed that this patient, with airway protection and in otherwise stable condition, should be monitored until symptoms resolved. Her symptoms proved to be self-limiting and resolved within 5 days.

In summary, we present a patient whose paradoxical reaction to midazolam was not reversed with flumazenil in the setting of urgent surgical management. Her symptoms persisted postoperatively but eventually were self-limiting. Although the etiology of these paradoxical reactions to benzodiazepines is still unclear, our case illustrates that general anesthesia with dexmedetomidine was insufficient to reverse this reaction, and a reversal agent could be considered to prevent prolonged postoperative hospital stays.
